# Abundance and distribution of archaeal acetyl-CoA/propionyl-CoA carboxylase genes indicative for putatively chemoautotrophic Archaea in the tropical Atlantic's interior

**DOI:** 10.1111/1574-6941.12073

**Published:** 2013-02-13

**Authors:** Kristin Bergauer, Eva Sintes, Judith van Bleijswijk, Harry Witte, Gerhard J Herndl, Tillmann Lueders

**Affiliations:** 1 Department of Marine Biology, Faculty Center of Ecology, University of ViennaVienna, Austria; 2 Department of Biological Oceanography, Royal Netherlands Institute for Sea ResearchDen Burg, Texel, The Netherlands

**Keywords:** chemoautotrophy, deep ocean, biotin carboxylase (*acc*A), Thaumarchaeota, DIC fixation, equatorial Atlantic, Romanche Fracture Zone

## Abstract

Recently, evidence suggests that dark CO_2_ fixation in the pelagic realm of the ocean does not only occur in the suboxic and anoxic water bodies but also in the oxygenated meso- and bathypelagic waters of the North Atlantic. To elucidate the significance and phylogeny of the key organisms mediating dark CO_2_ fixation in the tropical Atlantic, we quantified functional genes indicative for CO_2_ fixation. We used a Q-PCR-based assay targeting the bifunctional acetyl-CoA/propionyl-CoA carboxylase (*acc*A subunit), a key enzyme powering *inter alia* the 3-hydroxypropionate/4-hydroxybutyrate cycle (HP/HB) and the archaeal ammonia monooxygenase (*amo*A). Quantification of *acc*A-like genes revealed a consistent depth profile in the upper mesopelagial with increasing gene abundances from subsurface layers towards the oxygen minimum zone (OMZ), coinciding with an increase in archaeal *amo*A gene abundance. Gene abundance profiles of metabolic marker genes (*acc*A, *amo*A) were correlated with thaumarchaeal 16S rRNA gene abundances as well as CO_2_ fixation rates to link the genetic potential to actual rate measurements. *Acc*A gene abundances correlated with archaeal *amo*A gene abundance throughout the water column (*r*^2^ = 0.309, *P* < 0.0001). Overall, a substantial genetic predisposition of CO_2_ fixation was present in the dark realm of the tropical Atlantic in both *Archaea* and *Bacteria*. Hence, dark ocean CO_2_ fixation might be more widespread among prokaryotes inhabiting the oxygenated water column of the ocean's interior than hitherto assumed.

## Introduction

About two decades ago, molecular analyses have revealed that mesophilic *Archaea* [*Thaumarchaeota* (Brochier-Armanet *et al*., 2008), previously coined marine *Crenarchaeota* Group I, and Group II *Euryarchaeota*] are ubiquitously present in the global ocean.

The ability of some marine archaeal groups to incorporate dissolved amino acids (Ouverney & Fuhrman, 2000; Teira *et al*., 2006b) hints at a heterotrophic life mode. Compound-specific isotope analyses of thaumarchaeal lipids, however, revealed a chemoautotrophic lifestyle (Pearson *et al*., 2001; Wuchter *et al*., 2003). Pronounced differences in the Δ^14^C signature of thaumarchaeal DNA were found at a Pacific site at 670 and 900 m depth, indicating a preferential auto- and heterotrophic thaumarchaeal life mode, respectively (Hansman *et al*., 2009). Experimental work and genomic analysis using *Nitrosopumilus maritimus*, *Cenarchaeum symbiosum* and an enrichment culture obtained from the coastal North Sea (Könneke *et al*., 2005; Hallam *et al*., 2006a; Ingalls *et al*., 2006; Wuchter *et al*., 2006; Martens-Habbena *et al*., 2009) indicate that these members of the *Thaumarchaeota* are ammonia oxidizer (AOA) and hence chemolithoautotrophs.

The first step in ammonia oxidation is enzymatically catalysed by ammonia monooxygenase, and the encoding *amo*A gene is currently the most commonly used marker to determine the abundance of putatively ammonia-oxidizing prokaryotes in environmental studies. Numerous quantitative analyses have revealed that archaeal *amo*A genes dominate over bacterial *amo*A genes in soil as well as in marine and freshwater systems, tentatively suggesting that *Crenarchaeota* might be the main ammonia oxidizers in most environments (Treusch *et al*., 2005; Wuchter *et al*., 2006; Francis *et al*., 2007; Beman *et al*., 2008; Merbt *et al*., 2011). Hence, the oxidation of ammonia by AOA has been generally assumed to be the main energy source for dark carbon metabolism.

Apart from the prevalent pathway of carbon fixation, the Calvin–Bassham–Benson cycle (CBB), carried out by plants, algae and mostly photosynthetic bacteria, novel CO_2_ fixation pathways, such as the 3-hydroxypropionate/4-hydroxybutyrate (HP/HB) and dicarboxylate/4-hydroxybutyrate cycle (DC/4-HB), were found to exclusively operate in *Archaea* (Berg *et al*., 2007, 2010; Huber *et al*., 2008; Ramos-Vera *et al*., 2009). These metabolic pathways require unique and conserved enzymes that might serve as biomarkers for dark CO_2_ fixation. A key multifunctional enzyme of the HP/HB cycle, which is typically responsible for the synthesis of fatty acids in Bacteria, is the biotin-dependent acetyl-CoA/propionyl-CoA carboxylase complex (ACCase) (Moss & Lane, 1971; Chuakrut *et al*., 2003). Due to the lack of fatty acids in archaeal lipids, the enzyme is thought to mediate autotrophic carbon assimilation via the HP/HB cycle (Chuakrut *et al*., 2003; Hügler *et al*., 2003).

Additionally to dissolved inorganic carbon (DIC) fixation through autotrophic pathways, many carboxylases substantially contribute to the acquisition of carbon via assimilatory reactions as well as so-called anaplerotic reaction sequences (Werkman & Wood, 1942; Erb, 2011). While assimilatory carboxylases allow for a DIC ‘by-uptake’, anaplerotic reactions are ‘refilling’ the pools of tricarboxylic acid (TCA) cycle intermediates, intimately involved in the energy metabolism of cells. While anaplerotic CO_2_ fixation of heterotrophic *Bacteria* has been shown to occur in laboratory cultures, particularly associated with the uptake of labile substrate (Dijkhuizen & Harder, 1995), its contribution to the measured dark CO_2_ fixation in oceanic water remains enigmatic (Romanenko, 1964; Sorokin, 1993; Dijkhuizen & Harder, 1995; Alonso-Saez *et al*., 2010; Reinthaler *et al*., 2010).

The objective of this study was to determine the relative abundance and distribution of putatively chemoautotrophic prokaryotes in the dark realm of the Atlantic. We aimed at linking the genes indicative of chemoautotrophy and their abundance to dark DIC fixation. The abundances of *Archaea*-specific *acc*A genes were determined by Q-PCR and related to archaeal 16S rRNA and *amo*A gene abundances, as well as to DIC fixation rates. Based on our data, we conclude that meso- and bathypelagic prokaryotes might have a larger potential for autotrophy than assumed hitherto.

## Material and methods

### Sample collection

**Fig. 1:**
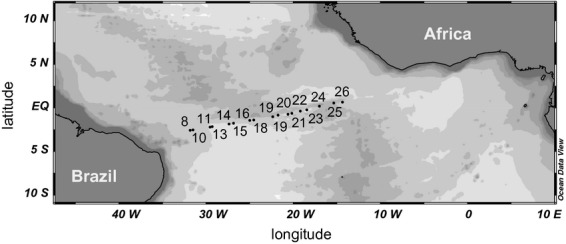
Map of the study area in the tropical Atlantic with the Stations 8–26 indicated by dots along a transect from the western to the eastern basin of the Atlantic through the Romanche Fracture Zone. Sampling was performed during the Archimedes-3 expedition in December 2007 to January 2008.

Water samples were collected from the major water masses throughout the water column (from the lower euphotic to the abyssopelagic zone) at 17 stations (Sts. 8–26) along a transect through the Romanche Fracture Zone (RFZ) in the tropical Atlantic during the Archimedes-3 cruise with RV *Pelagia* from December 2007 to January 2008 (Fig. 1). The physical and chemical characteristics of the sampled water masses are given in Table 1. Specifically, water was collected from the lower euphotic layer at 100 m depth, the South Atlantic Central Water (SACW) exhibiting an oxygen minimum, the Antarctic Intermediate Water (AAIW), the upper (uNADW), middle (mNADW) and lower North Atlantic Deep Water (lNADW) and the Antarctic Bottom Water (AABW) (Table 1). From these water masses, samples for prokaryotic variables (described below) as well as a suite of other microbial parameters (De Corte *et al*., 2011) were taken. Also, samples were collected to determine inorganic nutrient concentrations (Table 1) using standard spectrophotometric methods and a TRAACS autoanalyzer (Gordon *et al*., 1993).

**Table 1:** Physical characteristics and nutrient concentrations of the main water masses in the (sub)tropical North Atlantic sampled during the cruise Archimedes -3 at the stations 8–26

Water mass	Depth (m)	Temperature (°C)	Salinity	AOU (μmol kg^−1^)	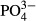 (μmol kg^−1^)	NO_3_^−^(μmol kg^−1^)	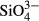 (μmol kg^−1^)
Subsurface	100	14.1–15.9	35.434–35.716	71.5–150.3	0.8–1.4	11.0–22.1	4.3–7.1
SACW	200–500	11.0–12.4	34.521–35.224	164.9–184.2	1.6–1.8	25.4–29.4	9.8–11.8
AAIW	500–1200	4.7–5.1	34.455–34.509	144.5–165.1	2.2–2.3	28.8–34.4	27.8–28.4
uNADW	1200–2500	3.8–4.0	34.976–34.982	70.2–82.0	1.27–1.33	19.3–20.2	16.2–17.7
mNADW	2500–3500	2.3–2.8	34.891–34.928	80.2–90.6	1.4–1.5	20.8–21.7	30.9–40.9
lNADW	3500–4250	2.0–2.4	34.870–34.902	76.1–87.7	1.3–1.5	19.6–21.4	32.6–45.2
AABW	4200–5500	0.9–1.4	34.749–34.779	112.9–121.4	1.8–2.0	27.2–29.3	82.7–94.5

Ranges are given for each water mass where samples were collected.

AOU, Apparent Oxygen Utilization; SACW, South Atlantic Central Water; AAIW, Antarctic Intermediate Water; uNADW, upper North Atlantic Deep Water; mNADW, middle North Atlantic Deep Water; lNADW, lower North Atlantic Deep Water; AABW, Antarctic Bottom Water.

### Picoplankton abundance determined by flow cytometry

Picoplankton collected from the different water masses were enumerated by flow cytometry. Water samples (2 mL) were fixed with 0.5% glutaraldehyde (final concentration), shock-frozen in liquid nitrogen for 5 min and stored at −80 °C. Immediately before analysis, the samples were thawed and the picoplankton cells stained with SYBR Green I at room temperature in the dark for 15 min and enumerated on a FACScalibur flow cytometer (Becton Dickinson) equipped with a 488-nm laser. Fluorescent microspheres (1 μm diameter, Molecular Probes) were added to all samples as an internal standard. Picoplankton cells were identified based on their characteristic right angle scatter vs. green fluorescence signature.

### Dark dissolved inorganic carbon fixation

^14^C-bicarbonate fixation in the dark was used to determine the bulk autotrophic activity of the prokaryotic community as described previously (Herndl *et al*., 2005). Briefly, 40 mL of seawater [samples and formaldehyde-fixed blanks (2% final conc.) each in triplicate] were spiked with ^14^C-bicarbonate (100 μCi; SA 54.0 mCi mmol^−1^; Amersham) and incubated in the dark at *in situ* temperature for 48–72 h. Subsequently, the samples were fixed with formaldehyde (2% final conc.), filtered onto 0.2-μm-pore-size filters (Millipore, polycarbonate, 25 mm filter diameter) supported by HAWP filters (Millipore, cellulose acetate, 0.45 μm pore-size) and rinsed three times with 10 mL of ultra-filtered seawater (30-kDa molecular weight cut-off). Thereafter, the filters were exposed to a fume of concentrated HCl for 12 h and placed in scintillation vials. Scintillation cocktail (8 mL Canberra-Packard Filter Count) was added, and after 18 h, the sample counted in a liquid scintillation counter (Tri-Carb 3100TR, PerkinElmer) on board RV *Pelagia*. The mean disintegrations per minute (DPM) of the formaldehyde-fixed blanks were subtracted from the mean DPM of the respective samples and the resulting DPM converted into DIC fixation rates taking into account the ambient DIC concentration measured by continuous-flow analysis (Stoll *et al*., 2001).

### DNA extraction

Ten litres of seawater were filtered through 0.2-μm Sterivex filter units (Millipore), and thereafter, 1.8 mL of lysis buffer (40 mM EDTA, 50 mM Tris-HCL, 0.75 M sucrose) was added to the filter cartridges, sealed at both ends with Parafilm and stored in the dark at −80 °C. Back in the laboratory, the Sterivex filter cartridges were cracked open and the lysis solution with the Sterivex filters transferred to sterile 50-mL centrifuge tubes. DNA extraction was carried out with the Mega Soil DNA extraction kit (MoBio laboratories, Carlsbad, CA) according to the manufacturer's protocol. Subsequently, isolated DNA was further concentrated (approximately 10 times) with a Centricon device (Millipore).

### Evaluating the primer set for detecting *acc*A genes

The annealing temperatures for the primer set Crena_529F/Crena_981R (Yakimov *et al*., 2009), specifically targeting archaeal acetyl-CoA carboxylase alpha subunit (*acc*A), were tested by a temperature gradient as follows: denaturation at 94 °C for 4 min; 35 cycles of denaturation at 94 °C for 40 s, annealing at 45–55 °C for 40 s and extension at 72 °C for 90 s, followed by an extension at 72 °C for 10 min and cooling at 4 °C. PCR products were checked on a 2% agarose gel. The optimal annealing temperature was at 51 °C for *acc*A primer set. PCR products of the expected size were obtained from enrichment cultures (*Nitrosopumilus maritimus*, *Sulfolobus solfataricus*, *Nitrosococcus oceani*, *Nitrosomonas europaea*), known to encode a bifunctional acetyl-CoA/propionyl-CoA carboxylase.

### Quantitative PCR (Q-PCR) *acc*A, archaeal *amo*A, archaeal 16S rRNA genes

All Q-PCRs were performed using an iCycler iQ 5 thermocycler (Bio-Rad) and i-Cycler iQ software (version 3.1, Bio-Rad). The reaction mixture was composed of 1 U of PicoMaxx high fidelity DNA polymerase (Stratagene), 2 μL of 10× PicoMaxx PCR buffer, 0.25 mM of each dNTP, 8 μg of BSA, 0.2 μM of each primer, 50 000 times diluted SYBR Green® (Invitrogen), and a final concentration of 10 nM of fluorescein, 3 mM of MgCl_2_, 1 μL of template DNA (average concentration 11.3 ng μL^−1^) and ultrapure sterile water (Sigma) was added to a final volume of 20 μL. All reactions were performed in iQ 96-well PCR plates (Bio-Rad) with optical sealing foils (Bio-Rad). The specificity of Q-PCRs was tested by electrophoresis on a 2% agarose gel and melting curve analysis. Standard curves were generated using serial dilutions of PCR products of known gene abundance (from 10^7^ to 10^1^ gene copies) obtained with the described primers (Table 2). The amount of DNA from the PCR products was determined spectrophotometrically (Nanodrop Technologies, Rockland, DE), and the gene abundance was calculated based on the fragment length and the DNA concentration. Ten-fold serial dilutions of the standard for the specific genes and no-DNA controls were run in triplicate with each plate. The following thermal cycling protocol was used: initial denaturation at 95 °C for 4 min; 41 cycles at 95 °C for 30 s, followed by the respective primer annealing temperature for 40 s, extension at 72 °C for 30 s and 80 °C for 25 s with readings taken between each cycle. To check for potential PCR artefacts, a melting curve analysis was performed at this point by monitoring SYBR Green fluorescence in the temperature ramp 60 to 94 °C with an increase of 0.5 °C and a hold for 1 s between each read.

**Table 2:** Q-PCR efficiencies and compilation of acetyl-CoA carboxylase alpha subunit–specific primers, 16S rRNA gene-targeting primers specific to Thaumarchaea and the archaeal *amo*A primer sets used to detect and quantify the respective genes

Target	Gene	Primer	Fragment length	Annealing temp. (°C)	Q-PCR Efficiency	Sequence (5′ to 3′)	Reference
*Thaumarchaea*	16S rRNA	MCGI-391-for MCGI-554-rev	122	61.0	84–96% (*r*^2^ = 0.983–0.997)	AAGGTTARTCCGAGTGRTTTC	Wuchter *et al*. (2006)
						TGACCACTTGAGGTGCTG	
Archaeal *amo*A _(1)_	*amo*A	Arch-*amo*A-for Arch-amoA-rev	256	58.5	98% (*r*^2^ = 0.998)	CTGAYTGGGCYTGGACATC	Wuchter *et al*. (2006)
						TTCTTCTTTGTTGCCCAGTA	
Archaeal *amo*A _(2)_	*amo*A	Arch-*amo*A-for Arch-amoA-rev NEW	256	58.5	101% (*r*^2^ = 0.999)	CTGAYTGGGCYTGGACATC	Wuchter *et al*. (2006), Konstantinidis *et al*. (2009)
						TTCTTCTTCGTCGCCCAATA	
*Thaumarchaea*	Biotin carboxylase, *acc*A	Crena_529-for Crena_981-rev	452	51	73% (*r*^2^ = 0.994)	GCWATGACWGAYTTTGTYRTAATG	Yakimov *et al*. (2009)
						TGGWTKRYTTGCAAYTATWCC	

The ‘total’ archaeal *amo*A gene abundance was calculated as the sum of archaeal *amo*A gene abundances yielded with the two primer sets Arch-*amoA*-for and Arch-*amoA*-rev as well as Arch-amoA-rev NEW (Table 2), assuming specific priming of distinct *amo*A gene types.

The efficiencies of Q-PCRs were 98% (*r*^2^ = 0.998) and 101% (*r*^2^ = 0.999) obtained with the archaeal *amo*A primers of Wuchter *et al*. (2006) and Konstantinidis *et al*. (2009), respectively. Amplification efficiency for the ACC*ase*-related primer pair was 73% (*r*^2^ = 0.994). The specificity of PCR products was confirmed by melting curve analysis and analyses of agarose gels. For *Thaumarchaeota*, primed with MCGI-391-for and MCGI-554-rev, Q-PCR efficiencies ranged between 84% and 96% (*r*^2^ = 0.983–0.997, Table 2).

### Statistical analyses

Statistics of Q-PCR data were performed with SigmaPlot, and linear regressions were calculated between archaeal *amo*A and *acc*A gene abundances.

## Results

### Hydrography

Specific water masses were sampled as shown by their distinct physical and chemical characteristics (Table 1). The highest apparent oxygen utilization (AOU) was detected in the SACW followed by AAIW and AABW (Table 1). The two lower NADW layers (mNADW and lNADW) were characterized by a higher concentration of silicate than the upper NADW reflecting a certain extent of mixing of the underlying AABW with its characteristically high silicate concentration with NADW (Table 1).

### Picoplankton and archaeal abundance

**Fig. 2:**
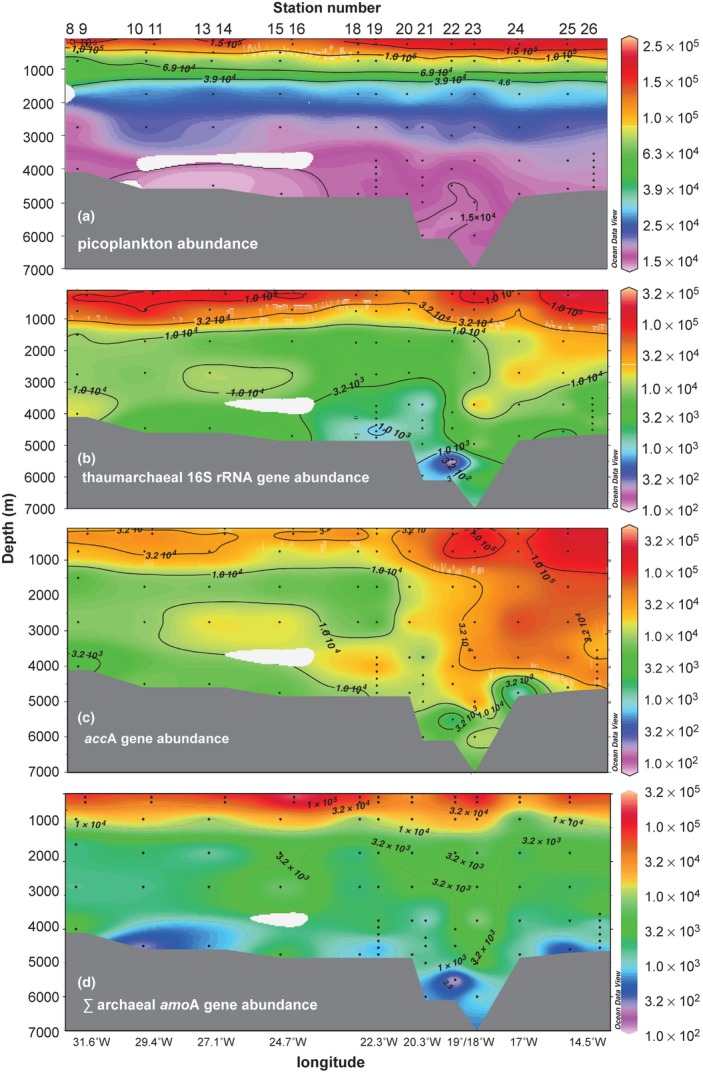
Cross-section through the Romanche Fracture Zone (down to 7150 m depth) showing (a) the distribution of picoplankton abundance determined by flow cytometry, (b) the abundance of the 16S rRNA genes of marine *Thaumarchaeota*, (c) the abundance of *acc*A-like genes and (d) the summed abundance of archaeal *amo*A genes determined by Q-PCR. Dots indicate positions where respective parameters were measured. For details on the depth range and the physical and chemical characteristics of the water masses, see Table 1.

Picoplankton abundance, determined by flow cytometry, decreased by one order of magnitude from the 100-m layer to the abyssopelagic waters with little lateral variability throughout the RFZ (Fig. 2a). Picoplankton abundance varied between 1.8 × 10^5^ and 3.5 × 10^5^ cells mL^−1^ at 100 m depth, 4.6 × 10^4^ and 2.4 × 10^5^ cells mL^−1^ in the mesopelagic waters, 1.6 × 10^4^ and 3.3 × 10^4^ cells mL^−1^ in the bathypelagic, and between 1.5 × 10^4^ and 3.8 × 10^4^ cells mL^−1^ in the abyssopelagic realm (Fig. 2a).

The 16S rRNA gene abundance of *Thaumarchaea* obtained by Q-PCR varied by 3 orders of magnitude over the entire depth range: between 1.5 × 10^4^ and 1.7 × 10^5^ mL^−1^ in the 100-m layer, 9.7 × 10^3^ and 3.9 × 10^5^ mL^−1^ in the mesopelagic, 5.9 × 10^2^ and 2.9 × 10^4^ mL^−1^ in the bathypelagic, and between 1.2 × 10^2^ and 1.6 × 10^4^ mL^−1^ in the abyssopelagic waters (Fig. 2b, Fig. S2). In the oxygen minimum zone (between 250 and 750 m depth), thaumarchaeal 16S rRNA gene abundance was significantly higher (Kruskal–Wallis, one-way anova; *P* ≤ 0.001, for 1750, 2750, 3500, 4500 and 6000 m) than in the waters below the O_2_ minimum layer. Overall, thaumarchaeal 16S rRNA gene abundances were significantly lower in the western than in the eastern part of the RFZ (anova on ranks, Kruskal–Wallis, *P* ≤ 0.001). However, no significant difference (anova on ranks, Dunn's method *P* ≥ 0.05) was detectable in the thaumarchaeal 16S rRNA gene abundance between the western and eastern part of the RFZ in the meso- and upper bathypelagic waters (200–2500 m, Fig. 2b).

### Distribution of carboxylase genes determined by Q-PCR

The abundance of acetyl-CoA/propionyl-CoA carboxylase alpha subunit (*acc*A) genes ranged between 1.1 × 10^4^ and 5.3 × 10^4^ mL^−1^ at 100 m depth, 1.1 × 10^4^ and 5.2 × 10^5^ mL^−1^ in the mesopelagic, 2.6 × 10^3^ and 4.9 × 10^4^ mL^−1^ in the bathypelagic, and between 7.6 × 10^2^ mL^−1^ and 4.8 × 10^4^ genes mL^−1^ in the abyssopelagic realm (Fig. 2c, Fig. S2). In the AAIW and SACW, the abundance of *acc*A genes was similar (anova on ranks, Kruskal–Wallis, Dunn's method, *P* ≥ 0.05) to thaumarchaeal 16S rRNA gene abundance (Fig. 2b and c).

**Fig. 3:**
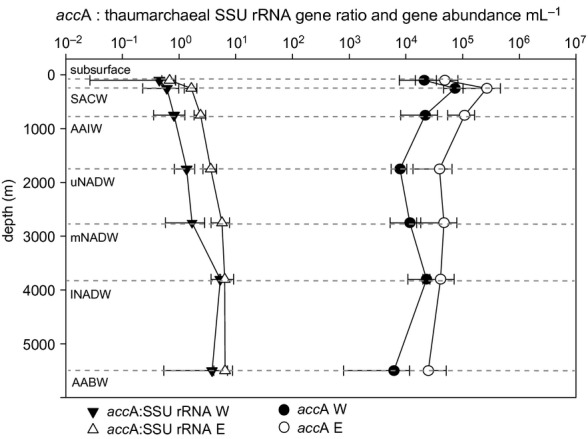
Depth profiles of *acc*A-like gene abundances and ratio of *acc*A-like gene abundance to 16S rRNA gene abundance of marine *Thaumarchaeota* in the western (W, Sts. 8–19) and eastern (E, Sts. 20–26) stations determined by Q-PCR. The mean ± SD gene abundance is given for each depth for the eastern and the western stations. Dashed lines delineate the water mass sampled, see Table 1.

Generally, *acc*A gene abundance was higher in the AAIW, SACW, NADW and the AABW in the eastern (Sts. 19–26) than in the western section of the RFZ (Figs 2c and 3). Total picoplankton, however, did not increase in abundance from west to east through the RFZ (Fig. 2a).

### Relation between archaeal *amo*A and *acc*A gene abundances

**Fig. 4:**
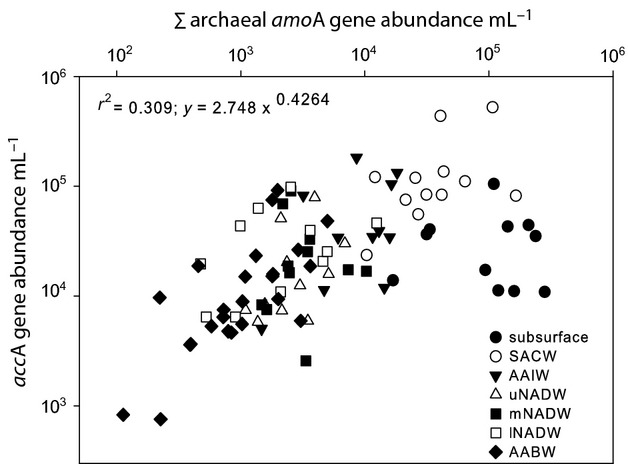
Relation of the abundance of archaeal *am*oA genes with *acc*A-like genes determined by Q-PCR. Different symbols indicate different water masses, see Table 1.

The *amo*A gene encoding the archaeal ammonia monooxygenase-α subunit was quantified as a proxy for putatively ammonia-oxidizing Archaea (Fig. 2d). The abundance of archaeal *amo*A genes obtained with both primer sets (Table 2) was summed and related to *acc*A gene abundance (Fig. 4). Archaeal *amo*A and *acc*A gene abundance were weakly related to each other over the entire depth range (*r*^2^ = 0.309, *P* < 0.0001, *n* = 91). Excluding the data of the 100 m-depth horizon, however, resulted in a tighter correlation (*r*^2^ = 0.407, *P* < 0.0001, *n* = 80) of archaeal *amo*A and *acc*A gene abundance than with the gene abundances of the 100 m layer included (Fig. 4).

### Relation between thaumarchaeal 16S rRNA and *acc*A gene abundance

The biotin carboxylase alpha subunit was significantly correlated with 16S rRNA gene abundances of *Thaumarchaeota*, excluding the samples from 100 m depth (*acc*A: *r*^2^ = 0.552, *P* < 0.0001, not shown). Including the gene abundance obtained in the 100 m layer resulted in a slightly weaker correlation with *acc*A genes (*r*^2^ = 0.493, *P* < 0.0001, not shown). The ratio of *acc*A/thaumarchaeal 16S rRNA gene abundance ranged between 0.6 and 5.9 from the subsurface to the bathypelagic layers, respectively (Fig. 3). In the O_2_ minimum zone (250–750 m), this ratio approached roughly unity and remained above 1 towards deeper layers (Fig. 3). No significant differences were found in the *acc*A/thaumarchaeal 16S rRNA gene ratio between the western and eastern part of the RFZ (Fig. 3; anova, Kruskal–Wallis, *P* > 0.05, respectively).

### Dark DIC fixation

Generally, prokaryotic dark DIC fixation rates increased from 4.3 ± 0.7 μmol C m^−3^ days^−1^ in the 100 m layer to 6.2 ± 4.2 μmol C m^−3^ days^−1^ at 250 m depth (Table 3, Supporting Information, Fig. S1). Below 250 m depth, DIC fixation rates were well below 1 μmol C m^−3^ days^−1^ (Table 3). In the central part of the RFZ (Sts. 16–19), higher dark DIC fixation rates were observed in the mesopelagic realm than in the marginal regions of the RFZ ranging from 6.7 to 14.1 μmol C m^−3^ days^−1^. Relating gene abundances to activity rates, only 30% of the variation in the CO_2_ fixation was explained by the summed abundance of archaeal *amo*A genes (not shown). A lower or similar explanatory power was found between DIC fixation rates and *acc*A (10%) and thaumarchaeal 16S rRNA (30%) gene abundances (data not shown).

**Table 3:** Mean ± SD of dissolved inorganic carbon (DIC) fixation rates in the dark and abundance of the *acc*A gene, the sum of archaeal *amo*A genes and the ratio of the sum of archaeal *amo*A genes: thaumarchaeal 16S rRNA gene. The standard deviations are given in parenthesis. For the gene abundance ratio, the mean values for the western and eastern parts of the RFZ are given separately in parenthesis. N.D. – not determined, SW – seawater

Depth [m]	Dark DIC fixation rate [μmol C m^−3^ days^−1^]	*acc*A [*n* × 10^3^ genes mL^−1^ SW]	∑ *amo*A [*n* × 10^3^ genes mL^−1^ SW]	Ratio (∑archaeal amoA):*Thaumarchaea* (western/eastern basin of the RFZ)
100	4.25 (± 0.7)	27.1 (± 16.0)	130.3 (± 87.0)	1.2 (1/0.6)
250	6.21 (± 4.16)	140.7 (± 134.8)	52.6 (± 45.3)	
500	0.11 (± 0.03)	N.D.	N.D.	N.D.
750	0.12 (± 0.13)	508.5 (± 40.2)	11.1 (± 5.0)	0.3 (0.2/0.3)
1750	0.1 (± 0.05)	16.0 (± 11.5)	3.0 (± 1.8)	0.4 (0.3/0.4)
2750	0.26 (± 0.34)	21.5 (± 13.0)	2.9 (± 1.7)	0.4 (0.3/0.3)
3500–4700	0.12 (± 0.17)	16.0 (± 14.6)	2.2 (± 2.6)	0.7 (0.2/0.5)

## Discussion

In this study, we related the abundance of genes encoding the archaeal biotin carboxylase and archaeal ammonia monooxygenase to dark DIC fixation rates, exploring the genetic potential for chemoautotrophy. The occurrence of archaeal *acc*A genes and their ubiquitous distribution throughout the pelagic realm of the open ocean are highly indicative of a chemoautotrophic lifestyle via the HP/HB cycle (Wuchter *et al*., 2006; Berg *et al*., 2007, 2010; Ward *et al*., 2007; Auguet *et al*., 2008; Pratscher *et al*., 2011) as archaeal lipids are devoid of fatty acids.

### DIC fixation in the dark ocean

Dark carbon metabolism and fixation rates in the oxygenated realm of the global ocean have been of interest for some time (Prakash *et al*., 1991), yet the organisms responsible for it remained ambiguous. Gradually, evidence is accumulating that DIC fixation in the dark ocean might be more prevalent than hitherto assumed (Auguet *et al*., 2008; Alonso-Saez *et al*., 2010; Hu *et al*., 2011; Varela *et al*., 2011). Measurements by Reinthaler *et al*. (2010) indicate dark DIC fixation rates in the meso- and bathypelagic waters of the North Atlantic amounts to about 15–53% of the export primary production. Comparative studies of prokaryotic heterotrophic biomass synthesis and DIC fixation in the dark realm of the North Atlantic revealed that DIC fixation is within the same order of magnitude as heterotrophic biomass production (Baltar *et al*., 2009; Reinthaler *et al*., 2010). Whether this DIC fixation is mainly due to autotrophic or heterotrophic microorganisms remains uncertain. Some recent studies emphasize the significance of anaplerotic CO_2_ conversion (Alonso-Saez *et al*., 2010; Lliros *et al*., 2011) in the context of microbial carbon cycling. Laboratory studies, however, provide contradictory evidence, showing that anaplerosis amounts to < 10% of organic carbon uptake by heterotrophic bacteria (Goldman *et al*., 1987; Dijkhuizen & Harder, 1995; Roslev *et al*., 2004). Anaplerosis, as reported by Doronina & Trotsenko (1985), appears to be stimulated by the presence of easily metabolizable organic carbon and consequently might play a minor role in the DIC fixation in deep waters (Reinthaler *et al*., 2010) where the organic carbon pool is largely refractory (Hansell *et al*., 2009; Jiao *et al*., 2010). A recent study indicates that members of the SAR324 cluster are ubiquitous present in the mesopelagic waters of the Atlantic and Pacific using RuBisCO as DIC acquisition enzyme (Swan *et al*., 2011). Hence, neither the extent of chemoautotrophy nor the potential metabolic pathways are probably completely known.

In this study, we determined the abundance of the archaeal *acc*A gene and used it as a marker for DIC fixation via the HP/HB cycle. This cycle functions in (micro) aerobic members of the crenarchaeal order *Sulfolobales* containing the acetyl-CoA/propionyl-CoA carboxylase for CO_2_ assimilation (Ishii *et al*., 1996; Menendez *et al*., 1999; Berg *et al*., 2007, 2010; Fuchs, 2011). The presence of genes encoding the key enzymes in the HP/HB cycle of *Thaumarchaeota* suggests that these and related abundant marine Archaea (Karner *et al*., 2001) may use a similar cycle. Key enzymes of the HP/HB cycle, including the acetyl-CoA/propionyl-CoA carboxylase, were unambiguously identified, whereas enzymes encoding genes of other autotrophic pathways were absent in the ammonia-oxidizing sponge symbiont *C. symbiosum* and the free-living *N. maritimus* (Könneke *et al*., 2005; Hallam *et al*., 2006a; Berg *et al*., 2007).

Q-PCR profiles of *acc*A genes assessed in the western and eastern part of the longitudinal transect of the RFZ revealed highly similar tendencies in their distribution along the depth strata (Fig. 3). The accuracy of determining the gene abundance of the *acc*A subunit by Q-PCR depends on the efficiency of the Q-PCR and a reliable standardization of the method. The observed Q-PCR efficiencies varied depending on the primer pair used and were lower amplifying the *acc*A gene (Yakimov *et al*., 2009) than other genes quantified in this study (Table 2). Determination of gene numbers and differences in efficiencies can be caused by a number of factors, such as initial extraction of nucleic acids, preparation and amplification of the standard curve template (Love *et al*., 2006), the presence of reverse transcriptase, reaction reagents, fragment size of the target gene and PCR inhibitors. *Acc*A primers, applied in DGGE analyses (Yakimov *et al*., 2009) as well as Q-PCR assays (Hu *et al*., 2011), yield rather long amplicons (Table 2), possibly impairing PCR efficiencies. However, amplification efficiencies were constant throughout our measurements and hence reproducible. We are confident that reliable estimates of the different gene abundances were achieved in this study. DNA extraction efficiencies were consistent, yielding DNA concentrations proportional to total cell counts (Fig. 2) within discrete depth layers (Table S2). Dilution series were performed *a priori* to check on PCR inhibitors empirically (see Supporting Information, Data S1). Variance in C_t_ values of triplicate reactions was higher when gene abundance was high and ranged between 10^2^ and 10^4^ gene copy numbers (Supporting Information, Table S1). Fierer *et al*. (2005), however, showed that varying DNA concentrations do not result in significant changes in gene abundances, supporting our conclusion of accurate gene numbers using the Q-PCR assay.

### Spatial variations in gene abundances

Prokaryotic abundance (Fig. 2) and dark DIC fixation (Table 3, Fig. S1) declined exponentially from surface to abyssopelagic layers as shown in previous studies from the North Atlantic (Reinthaler *et al*., 2006; Teira *et al*., 2006a; Varela *et al*., 2008). Also, a pronounced stratification in the composition of prokaryotic communities in the meso- and bathypelagic realm in the Atlantic and the Pacific has been reported (DeLong *et al*., 2006; Aristegui *et al*., 2009; Agogue *et al*., 2011). In this study, thaumarchaeal 16S rRNA gene abundance in the deep waters of the tropical Atlantic was within the range of abundances previously found (Varela *et al*., 2008; De Corte *et al*., 2009). However, thaumarchaeal 16S rRNA gene abundance profiles revealed a patchy spatial distribution in the upper mesopelagic layer and a more homogenous distribution below 1750 m depth (Fig. 2b). The percentage of thaumarchaeal 16S rRNA genes to total picoplankton counts (determined by flow cytometry) ranged between ∼7% and < 23% in the AABW, the oxygen minimum layer and in the lNADW. Similar abundances were also reported using CARD-FISH (catalyzed reporter deposition-fluorescence *in situ* hybridization) (Varela *et al*., 2008). Hence, independent of the quantification method used, a coherent pattern in the distribution of *Thaumarchaeota* in the different depth layers of the Atlantic is evident.

In the central-eastern part of the RFZ, the high abundance of thaumarchaeal 16S rRNA genes down to 1750 m depth (Fig. 2b, Sts. 15–23) coincided with high DIC fixation rates (10.5–14 μmol C m^−3^ days^−1^) and a high AOU (∼172 μmol O_2_ kg^−1^). While the high AOU may indicate an increased heterotrophic activity, the high DIC fixation rates are likely due to the activity of chemoautotrophs rather than anaplerotic reactions of heterotrophs because the autotrophic activity is similar to the measured heterotrophic prokaryotic biomass production (De Corte *et al*., 2010).

Correlating the abundance of biotin carboxylase α-subunit genes to the 16S rRNA gene abundance of *Thaumarchaeota*, ratios close to one were obtained in the central parts of the RFZ. The high abundance of *acc*A genes below 2000 m depth at Sts. 23 and 25 (Fig. 2c), however, is not reflected in enhanced DIC fixation rates (measured down to 4550 m depth, Fig. S1) or elevated concentrations of ammonia and/or nitrite (data not shown). This might indicate that the ecological function of *Crenarchaeota* is changing across the depth strata or alternative pathways of DIC fixation are followed. Similar dynamics have been shown by Grzymski *et al*. (2012), reporting seasonal shifts of chemolithoautotrophic organisms in surface waters of the Antarctic Peninsula.

A positive relation of thaumarchaeal 16S rRNA gene abundance and the concentration of ammonia (Wuchter *et al*., 2006; Kirchman *et al*., 2007; Varela *et al*., 2008) as well as nitrite (Teira *et al*., 2006a; Lam *et al*., 2007) has been reported for surface and mesopelagic waters, tentatively indicating chemolithoautotrophy of ammonia-oxidizing *Thaumarchaeota*. Dark DIC fixation rates normalized to *acc*A gene abundance varied over three to five orders of magnitude over all depths sampled (data not shown) reflecting either large variations in the expression of ACCase and/or a large variability in the enzymes responsible for CO_2_ fixation in the deep ocean.

Relating the gene abundance of archaeal *amo*A and thaumarchaeal 16S rRNA genes in the upper 250 m revealed a ratio of *accA*/thaumarchaeal 16S rRNA gene of ∼ 1.2 averaged over the whole transect of the RFZ (Table 3). Similar values were reported by Alonso-Saez *et al*. (2012) in polar oceans and Mincer *et al*. (2007) for the HOTS station in the Pacific, whereas Beman *et al*. (2008) and Wuchter *et al*. (2006) obtained *accA*/thaumarchaeal 16S rRNA gene ratios of about 2.5 and 2.8 in the Gulf of California and the North Atlantic, respectively. Significantly lower *accA*/thaumarchaeal 16S rRNA gene ratios (Table 3) were obtained from the lower meso- and bathypelagic waters of the RFZ, tentatively indicating that other energy sources than ammonia might be utilized in the lower meso- and bathypelagic waters.

Archaeal *amo*A and *acc*A gene abundances were only weakly correlated over the whole depth range (Fig. 4). A metagenomic survey by Hallam *et al*. (2006b) reported a gene ratio of *acc*A/*amo*A close to one in surface waters. In our study, the exclusion of the 100-m samples, however, resulted in a tighter relationship of archaeal *amo*A to *acc*A gene abundances. These differences might be caused by the specificity of the primers designed based on a few sequences (Yakimov *et al*., 2009), possibly failing to target *acc*A homologues of epipelagic *Archaea*. Data indicative of a vertical stratification of *Crenarchaeota* in a ‘shallow-’ and ‘deep-water clade’ have recently been suggested based on *acc*A gene diversity (Hu *et al*., 2011; Yakimov *et al*., 2011). A genomic and transcriptomic survey in the deep waters of the Gulf of California also revealed multiple populations of *Crenarchaeota* (Baker *et al*., 2012). Based on our data, the rather weak correlation between genes encoding the archaeal ammonia monooxygenase and acetyl-CoA carboxylase suggests that the HP/HB cycle might be fuelled at least to a certain extent by energy sources other than ammonia.

The overall ratio of the acetyl-CoA/propionyl-CoA carboxylase gene to thaumarchaeal 16S rRNA gene abundance supports the assumption that a major fraction of *Thaumarchaeota* is capable of fixing DIC via the HP/HB cycle. Assuming the *acc*A gene is present as a single copy in the crenarchaeal genome (Könneke *et al*., 2005; Hallam *et al*., 2006a), its abundances matches roughly those of thaumarchaeal cells in the SACW and AAIW (Fig. 3). In the water layers of the NADW and AABW, the ratio of *acc*A/thaumarchaeal 16S genes increased to 3.8 and 5.5, respectively. Highest *acc*A gene abundance (1.14 × 10^5^ genes mL^−1^) was obtained in the oxygen minimum zone (200–750 m depth) corresponding to the highest abundance of thaumarchaeal 16S rRNA genes. In a previous study, high *acc*A gene abundance (2.08 × 10^3^ ± 312 mL^−1^) was found at 3000 m depth in the Tyrrhenian Sea (western Mediterranean Sea) and a ratio of *acc*A/thaumarchaeal cells of about unity (Yakimov *et al*., 2009).

Overall, the gene abundances obtained by Q-PCR of archaeal *acc*A, archaeal *amo*A and thaumarchaeal 16S rRNA genes reveal similar trends of gene abundances with depth. The high abundance of thaumarchaeal 16S rRNA genes in the oxygen minimum layer might reflect the importance of *Thaumarchaeota* in the biogeochemical processes, particularly nitrification, occurring in this layer. However, also some groups of mesopelagic *Bacteria* such as members of the SAR324 or the SUP05 cluster contribute to autotrophy in the dark ocean (Swan *et al*., 2011).

Taken together, this study, along with some other recent work, demonstrates that there is a substantial genetic predisposition of DIC fixation present in the dark ocean. In this context, it adds to the emerging notion that dark ocean DIC fixation might be more important than hitherto assumed. The extent to which this DIC assimilation is due to anaplerotic reactions of heterotrophic prokaryotes or true autotrophy remains to be shown. Additional studies, including the quantification of other related genes and proteomic approaches, are needed to shed light on the modes of archaeal carbon metabolism. The sheer magnitude of DIC fixation, roughly equalling heterotrophic production in the North Atlantic deep waters, however, might indicate that the anaplerotic metabolism of heterotrophic prokaryotes is of minor importance and that autotrophic processes prevail in the DIC fixation pathway in the Atlantic's interior.

## Supporting Information

**Fig. S1.** Cross-section through the Romanche Fracture Zone (down to 4500 m depth) showing the horizontal and vertical variation of the prokaryotic dissolved inorganic carbon (DIC) fixation rates (μmol C m^−3^ days^−1^). Dots indicate positions where the respective parameter was measured.

**Fig. S2.** Depth profiles of mean abundances of archaeal *amo*A genes obtained with two different primer sets (Table 2), 16S rRNA gene abundance of marine *Thaumarchaeota* and *acc*A-like genes determined by Q-PCR. Dashed lines delineate the water mass sampled, see Table 1.

**Table S1.** Mean ± SD of raw (not normalized) gene abundances obtained with the different primers (Table 2) and their mean ± SD of Ct values calculated for 103 samples (in triplicate reactions) along a depth profile (Table 1).

**Table S2.** Mean ± SD of nucleic acid concentrations of 103 samples extracted along the Romanche fracture zone throughout the pelagic realm (100–7500 m).
