# Supplementary figures and images for: Abundance and distribution of archaeal acetyl-CoA/propionyl-CoA carboxylase genes indicative for putatively chemoautotrophic Archaea in the tropical Atlantic's interior
Source: FEMS Microbiol Ecol. 2013 Feb 13;84(3):461–73. doi: 10.1111/1574-6941.12073 (PMC3732383; doi:10.1111/1574-6941.12073)

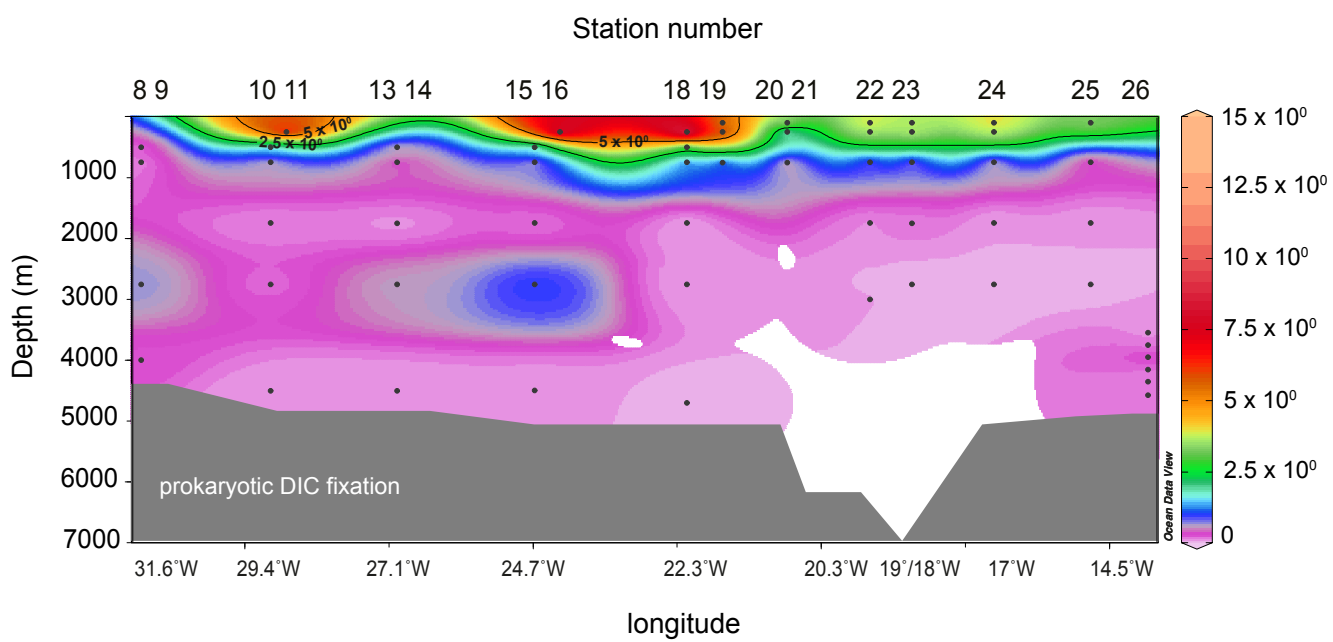

Fig. S1

Supplement: Supplementary file 1 [file fem0084-0461-SD1.pdf]

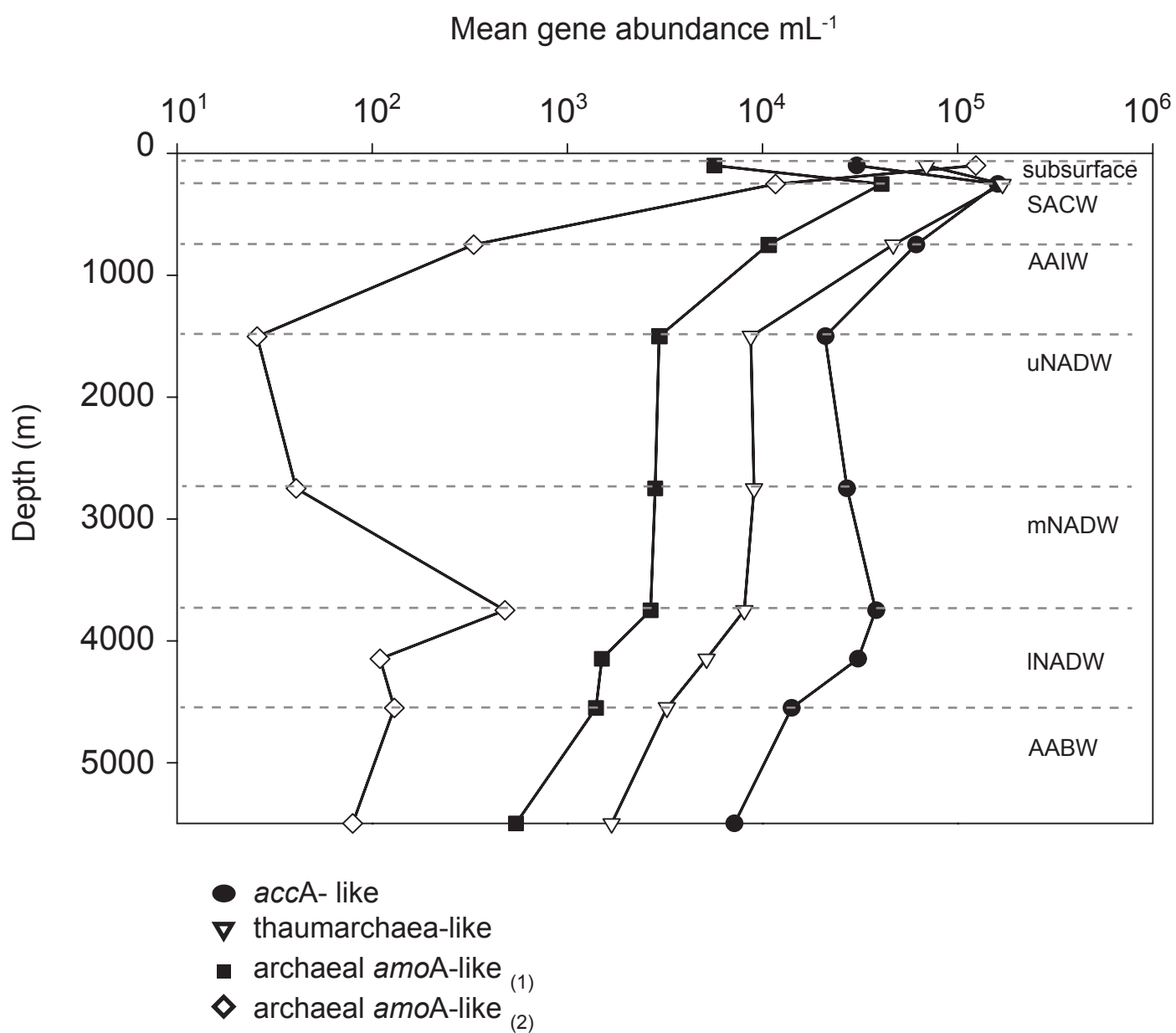

1

Fig. S2

Supplement: Supplementary file 2 [file fem0084-0461-SD2.pdf]
