# Supplementary material for: Abundance and distribution of archaeal acetyl-CoA/propionyl-CoA carboxylase genes indicative for putatively chemoautotrophic Archaea in the tropical Atlantic's interior
Source: FEMS Microbiol Ecol. 2013 Feb 13;84(3):461–73. doi: 10.1111/1574-6941.12073 (PMC3732383; doi:10.1111/1574-6941.12073)
Supplement: Supplementary file 3 [file fem0084-0461-SD3.pdf]

Table S1. Mean±SD of raw (not normalized) gene abundances obtained with the different primers (Table 2) and their mean±SD of Ct values calculated for 103 samples (in triplicate reactions) along a depth profile (Table 1).

**Depth (m)**	Water Mass	Archaeal *amoA* (2)	(+/-) Stdev. archaeal *amoA* (2)	Ct value archaeal *amoA* (2)	(+/-) Stdev. Ct value archaeal *amoA* (2)	Archaeal *amoA* (1)	(+/-) Stdev. archaeal *amoA* (1)	Ct value archaeal *amoA* (1)	(+/-) Stdev. Ct value archaeal *amoA* (1)	MCG I	(+/-) Stdev. MCG I	Ct value thaum-archaeal SSU	(+/-) Stdev. Ct value thaum-archaeal SSU	Archaeal *accA*	(+/-) Stdev. archaeal *accA*	Ct value archaeal *accA*	Stdev. Ct archaeal *accA*
**100**	Subsurface	2.18 x 10^05	4.51 x 10^03	18	0.11	8.54 x 10^06	3.51 x 10^04	17	0.22	3.61 x 10^06	5.77 x 10^03	22	0.11	3.08 x 10^04	2.42 x 10^04	29	2.39
		1.16 x 10^05	7.37 x 10^03	20	0.09	2.10 x 10^06	5.57 x 10^04	18	0.23	1.16 x 10^05	7.37 x 10^03	24	0.09	7.02 x 10^04	5.21 x 10^03	30	0.15
		4.16 x 10^05	1.57 x 10^04	20	0.06	1.07 x 10^07	5.77 x 10^04	18	0.10	4.16 x 10^05	1.57 x 10^04	24	0.06	1.73 x 10^05	3.46 x 10^03	27	0.20
		2.59 x 10^05	7.09 x 10^03	18	0.40	5.64 x 10^06	5.77 x 10^03	17	0.25	3.89 x 10^06	5.77 x 10^03	22	0.40	6.17 x 10^04	9.42 x 10^03	27	0.29
		1.37 x 10^06	2.23 x 10^04	19	0.55	2.46 x 10^07	5.77 x 10^04	18	0.22	9.85 x 10^06	6.93 x 10^04	22	0.55	1.76 x 10^05	6.60 x 10^04	26	0.78
		4.94 x 10^05	2.98 x 10^04	18	0.10	2.24 x 10^06	3.06 x 10^04	18	0.13	5.09 x 10^05	4.51 x 10^03	22	0.10	1.55 x 10^05	4.85 x 10^04	26	0.64
		3.60 x 10^05	1.53 x 10^03	20	0.07	1.19 x 10^07	5.77 x 10^04	18	0.08	3.66 x 10^05	3.79 x 10^03	24	0.07	1.47 x 10^05	7.42 x 10^03	27	0.67
		3.27 x 10^05	7.57 x 10^03	19	0.04	1.70 x 10^06	6.43 x 10^04	19	0.07	3.27 x 10^05	7.57 x 10^03	22	0.04	8.38 x 10^04	1.89 x 10^04	28	0.42
		6.45 x 10^04	3.36 x 10^03	20	0.09	7.91 x 10^06	3.46 x 10^04	17	0.09	1.23 x 10^06	5.77 x 10^03	24	0.09	3.89 x 10^04	1.50 x 10^04	27	0.69
		3.23 x 10^05	7.23 x 10^03	19	0.08	7.34 x 10^06	2.89 x 10^04	16	0.13	3.30 x 10^05	1.67 x 10^04	23	0.08	1.75 x 10^05	1.80 x 10^04	28	0.20
		8.36 x 10^05	4.63 x 10^04	19	0.10	2.25 x 10^07	2.89 x 10^04	18	0.21	5.45 x 10^06	1.00 x 10^04	24	0.10	6.08 x 10^04	1.65 x 10^04	28	1.96
**250**	SACW	4.53 x 10^06	2.08 x 10^03	14	0.07	2.19 x 10^05	1.56 x 10^04	23	0.14	4.43 x 10^06	3.61 x 10^04	19	0.07	6.86 x 10^05	7.00 x 10^03	23	0.22
		1.16 x 10^06	1.00 x 10^04	18	0.22	6.72 x 10^05	1.36 x 10^04	23	0.23	1.26 x 10^06	1.73 x 10^03	22	0.22	4.35 x 10^05	3.06 x 10^03	25	0.10
		4.07 x 10^06	3.46 x 10^04	16	0.25	1.84 x 10^06	4.93 x 10^04	22	0.26	4.90 x 10^06	2.65 x 10^04	20	0.25	1.56 x 10^06	1.44 x 10^04	22	0.38
		2.62 x 10^06	6.68 x 10^03	18	0.38	3.30 x 10^05	9.71 x 10^03	25	0.26	6.93 x 10^06	3.06 x 10^04	22	0.38	3.99 x 10^05	7.55 x 10^03	24	0.04
		5.27 x 10^05	3.43 x 10^04	18	0.10	4.38 x 10^05	1.15 x 10^03	20	0.30	5.27 x 10^05	3.43 x 10^04	22	0.10	2.63 x 10^05	1.70 x 10^04	27	0.44

		1.02 x 10^07	2.31 x 10^04	16	0.41	2.17 x 10^06	1.00 x 10^04	22	0.53	1.59 x 10^07	5.77 x 10^03	20	0.41	3.92 x 10^05	5.77 x 10^03	24	1.33
		1.54 x 10^06	5.77 x 10^03	17	0.29	1.60 x 10^06	1.15 x 10^04	19	0.10	1.54 x 10^06	1.53 x 10^04	21	0.29	8.39 x 10^05	1.15 x 10^04	25	0.11
		5.15 x 10^06	3.50 x 10^03	17	0.10	9.45 x 10^05	4.65 x 10^04	23	0.08	1.09 x 10^07	1.53 x 10^04	21	0.10	5.27 x 10^05	3.65 x 10^04	24	0.14
		1.54 x 10^06	1.53 x 10^03	15	0.39	4.58 x 10^05	7.64 x 10^03	22	0.10	9.19 x 10^06	5.77 x 10^02	19	0.39	2.45 x 10^05	4.04 x 10^03	24	0.30
		1.13 x 10^06	5.77 x 10^03	16	0.09	3.20 x 10^05	4.16 x 10^03	22	0.26	8.38 x 10^06	5.77 x 10^03	20	0.09	1.54 x 10^05	7.67 x 10^04	25	1.19
		1.13 x 10^06	1.00 x 10^04	16	0.22	4.43 x 10^05	2.08 x 10^03	22	0.12	8.55 x 10^06	6.66 x 10^03	20	0.22	2.90 x 10^05	1.55 x 10^04	24	0.10
**750**	AAIW	4.29 x 10^05	5.51 x 10^03	17	0.08	1.36 x 10^05	6.03 x 10^03	24	0.07	2.32 x 10^06	5.77 x 10^03	21	0.08	6.56 x 10^04	3.76 x 10^03	26	0.58
		6.28 x 10^05	7.51 x 10^03	18	0.09	8.38 x 10^03	9.26 x 10^02	27	0.17	6.28 x 10^05	7.51 x 10^03	22	0.09	3.26 x 10^05	8.60 x 10^03	27	0.08
		1.22 x 10^06	5.77 x 10^03	15	0.23	1.92 x 10^04	1.21 x 10^03	27	0.11	7.16 x 10^06	6.93 x 10^03	19	0.23	1.23 x 10^05	7.00 x 10^03	25	0.58
		4.64 x 10^05	8.08 x 10^03	20	0.26	4.47 x 10^03	1.09 x 10^03	32	0.42	1.43 x 10^06	6.44 x 10^03	24	0.26	6.95 x 10^04	7.72 x 10^03	28	0.23
		4.36 x 10^05	2.65 x 10^03	17	0.34	7.04 x 10^03	7.21 x 10^02	28	0.16	2.25 x 10^06	6.35 x 10^03	21	0.34	1.26 x 10^05	2.37 x 10^04	25	0.37
		2.85 x 10^05	3.98 x 10^03	19	0.20	4.42 x 10^03	5.23 x 10^02	28	0.20	2.85 x 10^05	3.98 x 10^04	23	0.20	1.87 x 10^05	6.08 x 10^03	28	0.33
		1.23 x 10^06	1.70 x 10^04	19	0.26	8.63 x 10^03	9.39 x 10^02	31	0.17	2.37 x 10^06	1.58 x 10^04	24	0.26	5.99 x 10^04	7.60 x 10^03	28	1.89
		1.41 x 10^06	6.81 x 10^03	16	0.08	8.28 x 10^03	6.11 x 10^02	29	0.15	1.43 x 10^06	2.89 x 10^03	21	0.08	2.64 x 10^05	6.86 x 10^03	26	0.48
		1.06 x 10^06	1.76 x 10^03	18	0.11	9.92 x 10^03	1.11 x 10^03	30	0.17	1.03 x 10^06	4.39 x 10^04	23	0.11	3.27 x 10^05	1.82 x 10^04	25	0.52
		1.12 x 10^06	1.00 x 10^03	18	0.02	1.66 x 10^04	1.11 x 10^04	29	1.00	1.12 x 10^06	5.77 x 10^03	23	0.02	4.26 x 10^05	4.36 x 10^03	25	0.09
		1.09 x 10^06	1.19 x 10^03	19	0.16	1.01 x 10^04	3.55 x 10^03	31	0.52	2.61 x 10^06	5.51 x 10^03	24	0.16	1.65 x 10^05	5.86 x 10^03	26	0.07
**1750**	uNADW	7.36 x 10^04	3.00 x 10^03	19	0.43	1.57 x 10^03	1.39 x 10^02	31	0.14	5.18 x 10^05	5.77 x 10^02	24	0.43	1.58 x 10^04	4.89 x 10^03	29	0.58
		2.67 x 10^05	1.44 x 10^04	19	0.25	2.17 x 10^03	1.72 x 10^02	29	0.13	3.01 x 10^05	5.20 x 10^04	24	0.25	1.51 x 10^05	2.34 x 10^04	28	0.29
		1.62 x 10^05	1.91 x 10^04	22	0.17	1.08 x 10^03	2.40 x 10^02	34	0.38	3.83 x 10^05	3.06 x 10^03	27	0.17	3.46 x 10^04	3.86 x 10^03	29	0.23
		2.43 x 10^05	3.93 x 10^04	21	0.24	1.50 x 10^03	8.96 x 10^01	34	0.09	4.47 x 10^05	2.08 x 10^03	26	0.24	2.10 x 10^04	3.98 x 10^03	29	0.36
		1.61 x 10^05	1.41 x 10^04	21	0.15	1.34 x 10^03	3.55 x 10^02	33	0.48	1.61 x 10^05	1.41 x 10^04	26	0.15	6.98 x 10^04	7.00 x 10^03	28	0.19

		1.51 x 10^05	1.06 x 10^04	19	0.10	2.31 x 10^03	2.53 x 10^02	30	0.18	6.74 x 10^05	7.21 x 10^03	23	0.10	2.63 x 10^04	8.96 x 10^03	28	0.72
		1.73 x 10^05	8.89 x 10^03	20	0.08	1.67 x 10^03	2.45 x 10^02	30	0.24	1.73 x 10^05	8.89 x 10^03	25	0.08	1.06 x 10^05	9.93 x 10^03	29	0.20
		2.97 x 10^05	3.93 x 10^04	21	0.19	2.54 x 10^03	5.65 x 10^02	33	0.35	8.43 x 10^05	5.62 x 10^03	25	0.19	6.23 x 10^04	9.89 x 10^02	28	0.32
		4.23 x 10^05	1.23 x 10^03	20	0.47	3.92 x 10^03	1.13 x 10^03	31	0.52	3.57 x 10^05	2.32 x 10^04	24	0.47	1.10 x 10^05	6.08 x 10^03	28	0.76
		5.40 x 10^05	7.03 x 10^03	18	0.20	2.51 x 10^03	4.08 x 10^02	31	0.30	6.00 x 10^05	1.80 x 10^04	22	0.20	7.64 x 10^04	3.42 x 10^03	27	0.37
		9.27 x 10^04	2.41 x 10^03	19	0.19	1.26 x 10^03	2.71 x 10^02	31	0.33	4.35 x 10^05	2.08 x 10^03	23	0.19	2.98 x 10^04	4.86 x 10^03	28	0.31
2750	mNADW	1.06 x 10^05	5.57 x 10^03	19	0.16	1.23 x 10^03	1.80 x 10^02	31	0.34	4.03 x 10^05	4.62 x 10^03	23	0.16	3.47 x 10^04	3.76 x 10^03	28	1.21
		4.03 x 10^05	1.15 x 10^04	20	0.04	4.63 x 10^03	1.69 x 10^02	32	0.55	7.63 x 10^05	8.14 x 10^03	24	0.04	4.82 x 10^04	1.74 x 10^04	27	0.80
		3.60 x 10^05	2.86 x 10^04	19	0.13	3.20 x 10^03	2.29 x 10^02	31	0.14	3.60 x 10^05	2.86 x 10^04	22	0.13	1.39 x 10^05	6.21 x 10^03	27	0.19
		2.02 x 10^05	6.51 x 10^03	20	0.05	6.25 x 10^03	6.10 x 10^02	27	0.16	2.02 x 10^05	6.51 x 10^03	23	0.05	1.65 x 10^05	1.08 x 10^04	28	0.28
		9.14 x 10^04	7.75 x 10^03	20	0.33	2.97 x 10^03	1.63 x 10^03	31	1.32	4.68 x 10^05	1.00 x 10^03	23	0.33	2.45 x 10^04	6.96 x 10^03	28	0.59
		2.26 x 10^05	2.68 x 10^04	20	0.17	4.93 x 10^03	8.92 x 10^02	28	0.28	2.34 x 10^05	1.82 x 10^04	23	0.17	2.07 x 10^05	1.86 x 10^04	28	0.17
		1.75 x 10^05	2.54 x 10^04	18	0.22	1.03 x 10^04	1.01 x 10^03	28	0.16	8.67 x 10^05	1.53 x 10^03	22	0.22	7.15 x 10^03	2.97 x 10^02	31	0.08
		2.22 x 10^05	3.65 x 10^04	21	0.23	2.91 x 10^03	4.19 x 10^02	33	0.22	5.19 x 10^05	2.51 x 10^04	25	0.23	7.43 x 10^04	1.36 x 10^04	28	0.35
		1.99 x 10^05	2.80 x 10^04	21	0.19	3.06 x 10^03	2.15 x 10^02	32	0.11	4.03 x 10^05	4.73 x 10^03	25	0.19	4.72 x 10^04	5.33 x 10^03	29	0.23
		1.68 x 10^05	5.69 x 10^03	18	0.43	2.29 x 10^03	2.61 x 10^02	30	0.19	9.52 x 10^05	5.03 x 10^03	22	0.43	7.82 x 10^04	3.93 x 10^03	26	0.10
		2.83 x 10^05	1.90 x 10^04	21	0.12	5.60 x 10^03	1.14 x 10^03	31	0.34	2.83 x 10^05	1.90 x 10^04	24	0.12	1.77 x 10^05	7.23 x 10^03	27	1.01
		1.03 x 10^06	4.39 x 10^04	24	0.19	1.68 x 10^03	3.32 x 10^02	31	0.32	4.28 x 10^05	2.19 x 10^04	28	0.07	3.83 x 10^04	3.92 x 10^03	31	0.20
3750	lNADW	4.09 x 10^05	4.40 x 10^03	18	0.19	8.54 x 10^03	2.39 x 10^03	29	0.51	4.49 x 10^05	8.96 x 10^03	22	0.19	9.52 x 10^04	4.77 x 10^03	27	0.14
		8.45 x 10^04	4.20 x 10^03	23	0.17	4.96 x 10^03	2.97 x 10^02	32	0.10	1.45 x 10^05	6.00 x 10^03	27	0.17	3.08 x 10^04	3.17 x 10^03	29	0.21
		2.27 x 10^05	2.12 x 10^04	19	0.15	5.08 x 10^03	7.87 x 10^02	30	0.30	2.17 x 10^05	5.29 x 10^03	23	0.15	6.05 x 10^04	6.08 x 10^03	28	0.20
		3.09 x 10^04	2.60 x 10^03	22	0.12	2.42 x 10^03	3.55 x 10^02	29	0.25	3.09 x 10^04	2.60 x 10^03	26	0.12	3.46 x 10^04	4.25 x 10^03	31	0.24

		5.40 x 10^05	1.61 x 10^04	19	0.05	3.22 x 10^05	1.85 x 10^04	24	0.11	5.33 x 10^05	5.69 x 10^03	23	0.05	2.01 x 10^05	6.77 x 10^03	27	1.13
		8.65 x 10^04	2.33 x 10^03	21	0.04	5.61 x 10^03	9.20 x 10^02	28	0.27	8.65 x 10^04	2.33 x 10^03	25	0.04	1.05 x 10^05	2.31 x 10^03	29	0.04
		4.95 x 10^04	3.62 x 10^03	23	0.12	1.47 x 10^03	6.80 x 10^02	33	0.83	4.95 x 10^04	3.62 x 10^03	27	0.12	3.13 x 10^04	3.00 x 10^03	30	0.19
		1.81 x 10^05	1.45 x 10^04	20	0.12	1.30 x 10^04	2.52 x 10^03	26	0.32	1.81 x 10^05	1.45 x 10^04	24	0.12	1.88 x 10^05	1.38 x 10^04	28	0.14
		7.46 x 10^04	2.72 x 10^03	21	0.16	4.75 x 10^03	3.84 x 10^02	28	0.13	7.12 x 10^04	7.44 x 10^03	25	0.16	8.34 x 10^04	6.62 x 10^03	29	0.15
		4.42 x 10^05	9.74 x 10^04	18	0.39	8.96 x 10^03	8.73 x 10^02	29	0.20	4.42 x 10^05	9.74 x 10^03	22	0.39	1.19 x 10^05	1.73 x 10^04	27	0.43
		2.31 x 10^05	8.96 x 10^03	21	0.07	1.92 x 10^04	5.55 x 10^03	29	0.53	2.31 x 10^05	8.96 x 10^03	25	0.07	1.46 x 10^05	6.11 x 10^03	27	0.29
**4500**	AABW	1.26 x 10^05	1.42 x 10^04	20	0.16	1.25 x 10^04	1.40 x 10^03	26	0.18	1.17 x 10^05	1.53 x 10^03	24	0.16	1.44 x 10^05	2.62 x 10^04	28	0.35
		2.91 x 10^05	2.19 x 10^04	19	0.13	1.15 x 10^04	1.83 x 10^03	28	0.31	2.91 x 10^05	2.19 x 10^03	23	0.13	8.33 x 10^04	1.97 x 10^03	28	0.33
		1.56 x 10^05	3.79 x 10^03	20	0.04	6.65 x 10^03	5.78 x 10^02	29	0.17	1.56 x 10^05	3.79 x 10^03	24	0.04	7.07 x 10^04	3.16 x 10^03	28	0.09
		1.32 x 10^05	3.79 x 10^03	20	0.04	1.43 x 10^04	1.80 x 10^03	26	0.20	1.32 x 10^05	3.79 x 10^03	24	0.04	1.70 x 10^05	7.21 x 10^03	28	0.08
		3.29 x 10^04	6.07 x 10^03	22	0.29	3.03 x 10^03	5.20 x 10^02	31	0.32	3.29 x 10^04	6.07 x 10^03	26	0.29	1.66 x 10^04	5.13 x 10^02	31	0.06
		7.46 x 10^04	9.98 x 10^03	21	0.42	5.18 x 10^03	4.36 x 10^02	30	0.16	7.46 x 10^04	9.98 x 10^02	25	0.42	3.87 x 10^04	1.89 x 10^03	29	0.09
		1.64 x 10^05	1.45 x 10^04	21	0.16	1.69 x 10^04	9.29 x 10^02	29	0.09	1.64 x 10^05	1.45 x 10^04	25	0.16	8.86 x 10^04	8.42 x 10^03	28	0.38
		1.52 x 10^05	1.36 x 10^04	22	0.13	1.58 x 10^04	1.00 x 10^03	30	0.17	3.57 x 10^05	1.15 x 10^03	26	0.13	1.66 x 10^04	3.40 x 10^03	29	0.42
		1.41 x 10^04	4.61 x 10^03	22	0.47	4.70 x 10^02	3.52 x 10^01	33	0.22	2.40 x 10^05	2.08 x 10^03	26	0.47	2.44 x 10^03	1.35 x 10^02	33	0.11
		6.11 x 10^04	2.26 x 10^03	20	0.06	4.66 x 10^03	6.66 x 10^01	29	0.07	4.34 x 10^05	3.21 x 10^03	24	0.06	2.93 x 10^04	1.88 x 10^03	28	0.13
		2.81 x 10^04	4.93 x 10^03	23	0.30	4.44 x 10^03	2.86 x 10^02	30	0.13	2.81 x 10^04	4.93 x 10^02	26	0.30	1.50 x 10^04	3.00 x 10^03	31	0.40
		2.63 x 10^04	1.40 x 10^03	23	0.08	4.12 x 10^03	7.19 x 10^02	28	0.27	2.63 x 10^04	1.40 x 10^03	26	0.08	3.13 x 10^04	7.77 x 10^02	31	0.05
		1.41 x 10^04	1.73 x 10^02	24	0.02	2.96 x 10^03	3.98 x 10^02	29	0.21	1.41 x 10^04	1.73 x 10^02	27	0.02	1.86 x 10^04	3.33 x 10^03	32	0.34
		1.12 x 10^05	3.21 x 10^03	22	0.05	1.17 x 10^04	2.46 x 10^03	30	0.43	2.55 x 10^05	5.77 x 10^02	26	0.05	3.35 x 10^04	2.94 x 10^03	29	0.17
		2.38 x 10^05	6.14 x 10^04	19	0.46	1.17 x 10^04	1.50 x 10^03	28	0.26	2.72 x 10^05	8.14 x 10^03	23	0.46	6.20 x 10^04	6.42 x 10^03	28	0.37

		6.62 x 10^04	3.28 x 10^04	21	0.75	5.93 x 10^03	9.75 x 10^02	30	0.34	6.62 x 10^04	3.28 x 10^04	25	0.75	2.17 x 10^04	2.08 x 10^03	30	0.20
		7.29 x 10^04	2.43 x 10^03	23	0.13	7.04 x 10^03	1.25 x 10^03	31	0.29	1.74 x 10^05	8.96 x 10^03	27	0.13	2.30 x 10^04	2.21 x 10^03	30	0.19
		9.18 x 10^04	1.16 x 10^04	22	0.21	1.01 x 10^04	2.47 x 10^03	30	0.43	9.18 x 10^04	1.16 x 10^04	26	0.21	4.48 x 10^04	8.44 x 10^03	30	0.35
		3.01 x 10^05	1.94 x 10^04	20	0.11	2.67 x 10^04	3.71 x 10^03	28	0.23	3.08 x 10^05	7.94 x 10^03	24	0.11	1.52 x 10^05	6.56 x 10^03	27	0.17
		1.71 x 10^05	5.14 x 10^04	20	0.67	9.87 x 10^03	4.04 x 10^02	29	0.08	1.57 x 10^05	6.29 x 10^04	24	0.67	7.01 x 10^04	3.90 x 10^03	28	0.11
**6000**	AABW	1.01 x 10^04	2.36 x 10^03	26	0.41	1.16 x 10^03	2.63 x 10^02	33	0.38	1.01 x 10^04	2.36 x 10^03	30	0.41	4.14 x 10^03	1.65 x 10^02	34	0.08
		7.58 x 10^04	5.25 x 10^04	23	1.80	1.38 x 10^04	2.16 x 10^03	29	0.24	7.58 x 10^04	5.25 x 10^02	27	1.80	6.23 x 10^04	5.27 x 10^02	29	0.17
		6.02 x 10^04	2.71 x 10^03	21	0.08	5.82 x 10^03	2.90 x 10^02	30	0.09	6.02 x 10^04	2.71 x 10^03	25	0.08	3.41 x 10^04	2.96 x 10^03	30	0.17
		4.11 x 10^04	2.16 x 10^03	24	0.09	6.07 x 10^03	1.88 x 10^03	31	0.51	4.11 x 10^04	2.16 x 10^03	28	0.09	2.16 x 10^04	1.24 x 10^03	31	0.11
