# Supplementary material for: Abundance and distribution of archaeal acetyl-CoA/propionyl-CoA carboxylase genes indicative for putatively chemoautotrophic Archaea in the tropical Atlantic's interior
Source: FEMS Microbiol Ecol. 2013 Feb 13;84(3):461–73. doi: 10.1111/1574-6941.12073 (PMC3732383; doi:10.1111/1574-6941.12073)
Supplement: Supplementary file 4 [file fem0084-0461-SD4.pdf]

Table S2. Mean±SD of nucleic acid concentrations of 103 samples extracted along the Romanche fracture zone throughout the pelagic realm (100 – 7500m).

| Depth [m] | Average DNA concentration (ng/µl) | SD |
|---|---|---|
| 100 | 21.5 | ± 8.10 |
| 250 | 15.0 | ± 3.96 |
| 750 | 8.6 | ± 2.23 |
| 1750 | 6.9 | ± 2.58 |
| 2750 | 8.3 | ± 3.57 |
| 3750 | 8.2 | ± 3.76 |
| 4000-7000 | 7.3 | ± 3.71 |
