# Supplementary material for: Abundance and distribution of archaeal acetyl-CoA/propionyl-CoA carboxylase genes indicative for putatively chemoautotrophic Archaea in the tropical Atlantic's interior
Source: FEMS Microbiol Ecol. 2013 Feb 13;84(3):461–73. doi: 10.1111/1574-6941.12073 (PMC3732383; doi:10.1111/1574-6941.12073)
Supplement: Supplementary file 9 [file fem0084-0461-SD9.docx]

**Supplementary methods**

**Sample collection**

Samples from distinct water masses, identified based on their temperature and salinity characteristics and inorganic nutrient concentrations were taken with 12 L Niskin bottles mounted in a conductivity-temperature-depth (CTD) rosette sampler holding sensors for conductivity, temperature, depth, chlorophyll fluorescence, and optical backscattering.

**DNA extraction.**

Genomic DNA was extracted as described in the manuscript. DNA concentrations were quantified by A_260_ absorbance determinations conducted on a spectrophotometer. DNA concentrations were fairly consistent within specific depth layers and mean (±SD) DNA concentrations are summarized in Table S2. A total of 103 environmental samples with cell numbers varying by one order of magnitude were extracted. Despite the varying cell abundance with depth, the average DNA concentration varied only by a factor of less than 3 and was similar between 750 m and 7000 m depth (Table S2).

Per sample, a volume of 100-150µl DNA was extracted and the Q-PCR was performed in triplicate reactions per sample. PCR inhibitors present in extracted nucleic acids from natural environments influence Q-PCR efficiencies ([Stults*, et al.*, 2001](#_ENREF_1)). Hence, dilution series were performed to determine amplification efficiencies.

C_t_ values and their equivalent gene numbers (Table S1) are given for all Q-PCR assays in order to determine the lower limits of detection for each reaction. Internal standard curves were constructed by amplification of target genes of mixed communities and plotting the resulting C_t_ values against target DNA concentrations. DNA standards consisted of PCR amplicons obtained from environmental samples produced by the different primer pairs (Table 2). Triplicate reactions were pooled and purified with the Ultra Clean PCR clean-up kit (MoBIO laboratories, Carlsbad, CA, USA) and quantified by averaging three replicate A_260_ absorbance determinations conducted on a spectrophotometer. Hence, amplification efficiencies of standards and environmental samples should be equal among different Q-PCR assays, although it cannot be ruled out completely that the amplification efficiency of PCR amplicons might be different from genes of a mixed community.
